# Phase I Trial of First-in-Class ATR Inhibitor M6620 (VX-970) as Monotherapy or in Combination With Carboplatin in Patients With Advanced Solid Tumors

**DOI:** 10.1200/JCO.19.02404

**Published:** 2020-06-22

**Authors:** Timothy A. Yap, Brent O’Carrigan, Marina S. Penney, Joline S. Lim, Jessica S. Brown, Maria J. de Miguel Luken, Nina Tunariu, Raquel Perez-Lopez, Daniel Nava Rodrigues, Ruth Riisnaes, Ines Figueiredo, Suzanne Carreira, Brian Hare, Katherine McDermott, Saira Khalique, Chris T. Williamson, Rachael Natrajan, Stephen J. Pettitt, Christopher J. Lord, Udai Banerji, John Pollard, Juanita Lopez, Johann S. de Bono

**Affiliations:** ^1^ Drug Development Unit, Royal Marsden Hospital, London, United Kingdom; ^2^ The Institute of Cancer Research, London, United Kingdom; ^3^ Vertex Pharmaceuticals, Boston, MA; ^4^ Breast Cancer Now Toby Robins Research Centre, The Institute of Cancer Research, London, United Kingdom; ^5^ CRUK Gene Function Laboratory, The Institute of Cancer Research, London, United Kingdom; ^6^ Vertex Pharmaceuticals, Oxfordshire, United Kingdom

## Abstract

**PURPOSE:**

Preclinical studies demonstrated that ATR inhibition can exploit synthetic lethality (eg, in cancer cells with impaired compensatory DNA damage responses through ATM loss) as monotherapy and combined with DNA-damaging drugs such as carboplatin.

**PATIENTS AND METHODS:**

This phase I trial assessed the ATR inhibitor M6620 (VX-970) as monotherapy (once or twice weekly) and combined with carboplatin (carboplatin on day 1 and M6620 on days 2 and 9 in 21-day cycles). Primary objectives were safety, tolerability, and maximum tolerated dose; secondary objectives included pharmacokinetics and antitumor activity; exploratory objectives included pharmacodynamics in timed paired tumor biopsies.

**RESULTS:**

Forty patients were enrolled; 17 received M6620 monotherapy, which was safe and well tolerated. The recommended phase II dose (RP2D) for once- or twice-weekly administration was 240 mg/m^2^. A patient with metastatic colorectal cancer harboring molecular aberrations, including ATM loss and an *ARID1A* mutation, achieved RECISTv1.1 complete response and maintained this response, with a progression-free survival of 29 months at last assessment. Twenty-three patients received M6620 with carboplatin, with mechanism-based hematologic toxicities at higher doses, requiring dose delays and reductions. The RP2D for combination therapy was M6620 90 mg/m^2^ with carboplatin AUC5. A patient with advanced germline *BRCA1* ovarian cancer achieved RECISTv1.1 partial response and Gynecologic Cancer Intergroup CA125 response despite being platinum refractory and PARP inhibitor resistant. An additional 15 patients had RECISTv1.1 stable disease as best response. Pharmacokinetics were dose proportional and exceeded preclinical efficacious levels. Pharmacodynamic studies demonstrated substantial inhibition of phosphorylation of CHK1, the downstream ATR substrate.

**CONCLUSION:**

To our knowledge, this report is the first of an ATR inhibitor as monotherapy and combined with carboplatin. M6620 was well tolerated, with target engagement and preliminary antitumor responses observed.

## INTRODUCTION

The DNA damage response (DDR) provides cellular defense against DNA damage and is regulated by apical kinases ATM (ataxia-telangiectasia mutated) and ATR (ATM and Rad3 related).^1^ ATM is recruited to double-strand breaks (DSBs), whereas ATR is recruited to single-stranded DNA (ssDNA) coated with RPA. ssDNA can arise from DSB processing or stalled replication forks (replication stress [RS]). RS can occur when replication forks encounter unresolved DNA lesions or the replication rate outpaces the nucleotide supply.^2^ Both events are common in cancer (eg, from chemotherapy or oncogenes that drive rapid unscheduled proliferation).^2^ Once activated, ATM and ATR signal DNA damage to cell cycle checkpoints and promote homologous recombination (HR) repair.^3^ Despite the importance of the DDR, many tumors carry ATM pathway aberrations, placing a reliance on the ATR pathway for survival.^4,5^ Preclinical studies demonstrated that ATR inhibition lethally sensitizes many tumors with ATM pathway defects to chemotherapy-induced DNA damage.^5^ ATR inhibition is also effective as monotherapy in some cancer cells with ATM loss or other key DDR aberrations or tumors that express oncogenes, which drive high RS.^6^

**CONTEXT**

**Key Objective**
Can ATR inhibition lead to single-agent antitumor activity and enhance the effects of carboplatin chemotherapy safely in patients with advanced solid tumors, including those with relevant molecular aberrations?
**Knowledge Generated**
The ATR inhibitor M6620 was well tolerated, with anecdotal single-agent durable RECISTv1.1 complete response in a patient with metastatic colorectal cancer harboring molecular aberrations, including ATM loss and an *ARID1A* mutation. M6620 was well tolerated in combination with carboplatin chemotherapy at biologically active doses, with the observation of clinical activity in patients with advanced solid tumors, including a patient with platinum-refractory and PARP inhibitor–resistant germline *BRCA1* ovarian cancer.
**Relevance**
These findings provide early clinical proof of concept that ATR inhibitors may represent a novel antitumor strategy as monotherapy or in combination with carboplatin chemotherapy in patients with relevant molecular aberrations, including those who are platinum refractory or PARP inhibitor resistant, which are areas of unmet clinical need.

M6620 (formerly VX-970) is a first-in-class potent ATP-competitive ATR inhibitor with > 100-fold selectivity over related kinases (eg, DNA-PK and ATM).^7^ In preclinical studies, cells defective in ATM signaling were acutely sensitive to M6620 combined with genotoxic chemotherapy.^7^ In mouse xenograft models, M6620 10-20 mg/kg administered intravenously demonstrated synergistic antitumor efficacy with multiple chemotherapeutics, including platinum-based chemotherapy, often resulting in marked tumor growth inhibition or regression.^7,8^ These studies demonstrated that optimal combination efficacy was achieved when ATR inhibition was administered after chemotherapy.^7^

On the basis of these preclinical data, we conducted a phase I dose-escalation trial to determine safety, tolerability, maximum tolerated dose (MTD), pharmacokinetics, and antitumor activity of M6620 monotherapy and combined with carboplatin in patients with advanced solid tumors. An important objective was to assess the pharmacodynamic effects of M6620 combined with carboplatin. Next-generation sequencing (NGS) of genetic aberrations and ATM immunohistochemistry (IHC) were conducted on archival and/or fresh tumor specimens, when available, to assess predictive markers of response.

## PATIENTS AND METHODS

### Patient Population

Patients age ≥ 18 years with histologically confirmed advanced solid tumors refractory to standard therapy and RECISTv1.1 measurable disease^9^ were eligible (Data Supplement).

### Study Design

This phase I, open-label trial investigated the safety and tolerability of M6620 as monotherapy and combined with carboplatin in patients with refractory solid tumors (Data Supplement). The study was designed by academic investigators (T.A.Y. and J.S.d.B.) at the Royal Marsden Hospital (RMH) and representatives of Vertex Pharmaceuticals and conducted at RMH.

Patients received M6620 monotherapy once weekly in 21-day cycles using single-patient dose-escalation cohorts, expanding to 3 + 3 cohorts if Common Terminology Criteria for Adverse Events version 4.0^10^ grade (G) ≥ 2 toxicities were observed. The starting dose level (DL) was 60 mg/m^2^ and was informed by safety results from study VX12-970-001 (ClinicalTrials.gov identifier: NCT02157792). For the twice-weekly schedule, M6620 was administered on days 1 and 4, 8 and 11, and 15 and 18 of a 21-day cycle using a 3 + 3 design, with the starting dose determined by the highest tolerated dose from the once-weekly schedule.

### Pharmacodynamics

Timed tumor biopsies were taken from 12 patients with accessible disease at predefined time points in cycle 1. Approximately 22 hours after administration of carboplatin on day 1, the first biopsy sample was collected. M6620 was administered, and approximately 2 hours later, the second biopsy was collected (preferably from the same site). The 2-hour time point was based on preclinical data that showed that ATR-dependent CHK1 Ser345 phosphorylation is lost a few hours after treatment with an ATR inhibitor.^11^ After collection, tumor tissues were formalin fixed and paraffin embedded (FFPE). FFPE blocks were sectioned and analyzed for pCHK1 staining by IHC using the rabbit anti–p-Chk1 antibody (Cell Signaling Technology; Danvers, MA; product number 2348).

## RESULTS

### Patient Characteristics

**FIG 1.**
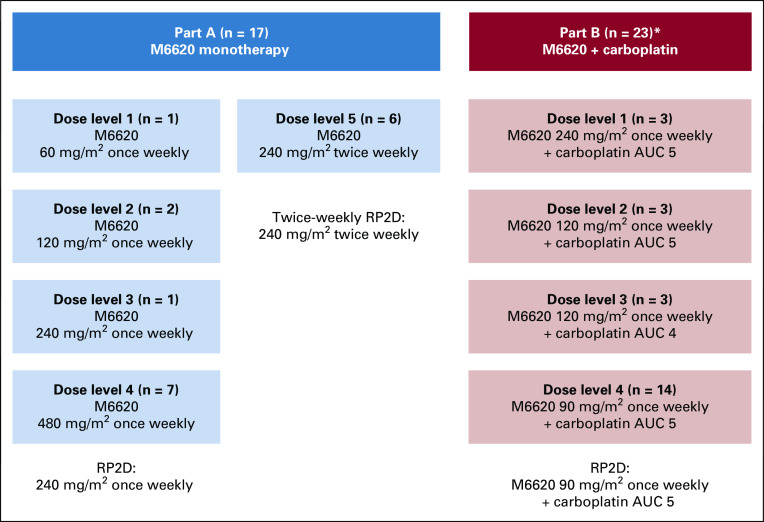
Patient flow diagram. AUC, area under the curve; RP2D, recommended phase II dose. (*) Part B started with a dose-escalation design, but dose reductions were required because of toxicities.

Overall, 40 patients were entered into this study and included in the safety analysis (Fig 1). All 40 patients received ≥ 1 M6620 dose and were considered evaluable for toxicity; their characteristics are listed in Table 1. Baseline mutations and ATM IHC results are reported for assessed patients in the Data Supplement.

**TABLE 1.**
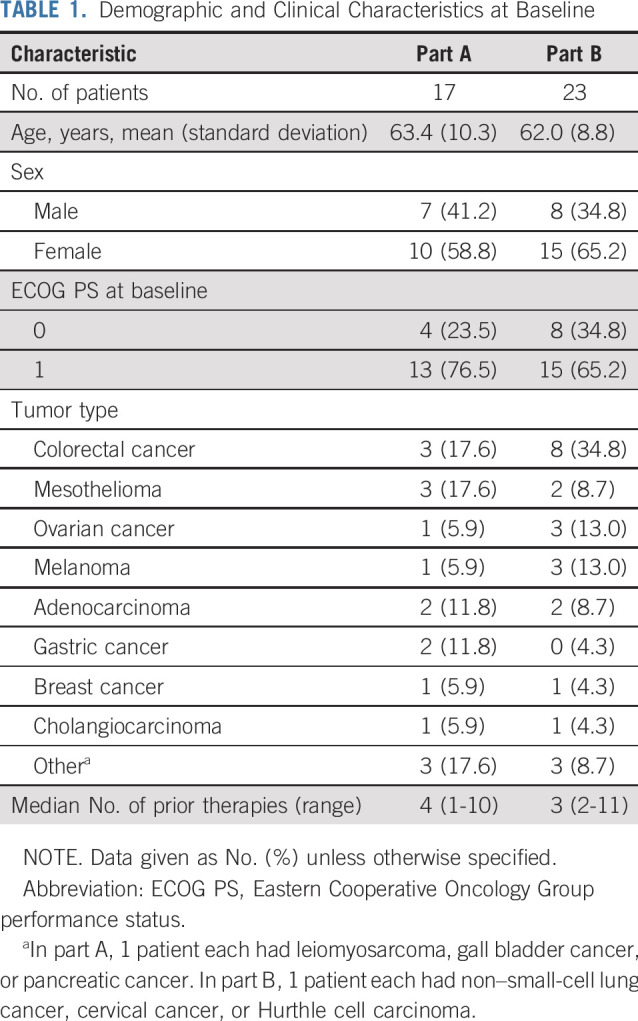
Demographic and Clinical Characteristics at Baseline

### Dose Escalation and Toxicity

#### M6620 monotherapy.

Once-weekly dose escalation proceeded through DLs of 60 mg/m^2^ (n = 1), 120 mg/m^2^ (n = 2), 240 mg/m^2^ (n = 1), and 480 mg/m^2^ (n = 7; Fig 1; Data Supplement), with no dose-limiting toxicities (DLTs) observed. Dose escalation was expanded at 480 mg/m^2^ once weekly and capped at this dose, determined to be the maximal administered dose (MAD), primarily because of the large infusion volumes required and because this dose exceeded the human equivalent dose from mouse efficacy studies (approximately 60 mg/m^2^). The once-weekly recommended phase II dose (RP2D) was selected as 240 mg/m^2^ because of antitumor activity at lower doses (eg, RECISTv1.1 complete response [CR] at 60 mg/m^2^) and prohibitive infusion volumes at 480 mg/m^2^. A twice-weekly schedule was assessed in a cohort of 6 patients at 240 mg/m^2^ (Fig 1; Data Supplement); this dose was well-tolerated, with no DLTs observed, establishing the twice-weekly RP2D at 240 mg/m^2^.

M6620 monotherapy was generally well tolerated for once-weekly and twice-weekly dosing (Tables 2 and 3). The most common all-grade, treatment-related, treatment-emergent adverse events (TEAEs) included flushing (23.5%), nausea (11.8%), pruritus (11.8%), headache (11.8%), and infusion-related reactions (11.8%). There were no clinically relevant trends in electrocardiogram assessments.

**TABLE 2.**
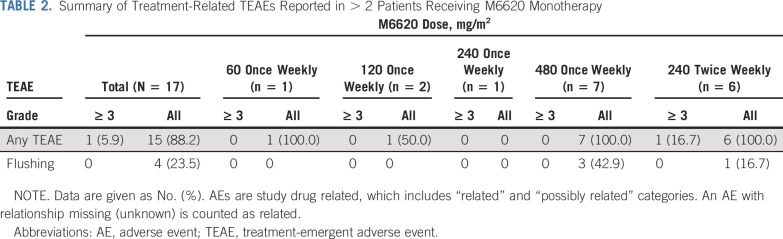
Summary of Treatment-Related TEAEs Reported in > 2 Patients Receiving M6620 Monotherapy

**TABLE 3.**
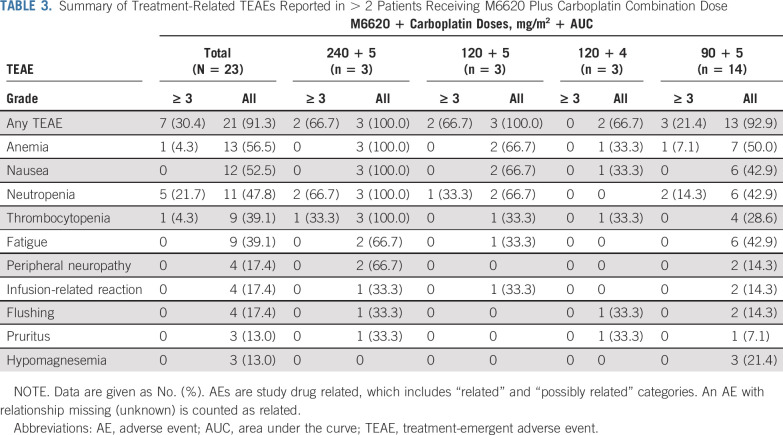
Summary of Treatment-Related TEAEs Reported in > 2 Patients Receiving M6620 Plus Carboplatin Combination Dose

#### Combination M6620 therapy.

The starting M6620 dose combined with carboplatin area under the curve (AUC) 5 was 240 mg/m^2^, 50% of the monotherapy MAD (Fig 1; Data Supplement). Three patients were treated at DL1, with DLTs of neutropenia, thrombocytopenia, and lower respiratory tract infection in a heavily pretreated patient with advanced cholangiocarcinoma. Dose reductions were required for all 3 patients because of G3/4 treatment-related neutropenia; although the neutropenia did not constitute a DLT by protocol, all patients required delays in the start of cycle 2. The trial safety monitoring committee elected to de-escalate M6620 to 120 mg/m^2^, maintaining carboplatin AUC 5 (DL2). With DL2, 1 patient experienced a DLT of G3 hypersensitivity, and 2 of 3 patients required dose reductions due to G3/4 treatment-related neutropenia and delays in commencing cycle 2. DL3 explored carboplatin AUC 4 with M6620 120 mg/m^2^. At DL3, no DLTs or G3/4 hematologic toxicities were observed, and all 3 patients received treatment without dose delays, interruptions, or reductions. DL4 of carboplatin AUC 5 with M6620 90 mg/m^2^ was explored, and 1 DLT of febrile neutropenia was observed in a patient with metastatic breast cancer who recovered without sequelae; she was heavily pretreated with 11 lines of cytotoxic therapies. The cohort was subsequently expanded to include 10 additional patients (14 total patients), and no other DLTs or G3/4 hematologic toxicities were observed. The combination therapy RP2D was established at carboplatin AUC 5 on day 1, with M6620 90 mg/m^2^ on days 2 and 9 of a 21-day cycle. This is higher than the estimated human dose (body surface area conversion) equivalent to M6620 10-20 mg/kg administered intravenously in mouse xenografts, in which efficacy was observed in combination with cytotoxic chemotherapy, including platinum agents.^8,12^

The most common all-grade TEAEs observed with combination therapy were mechanism-based hematologic toxicities, including neutropenia (47.8% [G3/4, 26.1%]), thrombocytopenia (39.1% [G3/4, 4.3%]), and anemia (56.5% [G3/4, 4.3%]). Only 1 nonhematologic G3/4 TEAE was observed: G3 hypersensitivity reaction at DL2. Other common all-grade nonhematologic TEAEs included nausea (52.5%) and fatigue (47.8%). There were no clinically relevant trends in electrocardiogram assessments. All TEAEs were manageable by standard guidelines and resolved without sequelae after discontinuation of the drug(s). No G5 treatment-related TEAEs were observed during the study.

### Pharmacokinetic Data

After once-weekly M6620 monotherapy, M6620 exposures increased in a dose-dependent manner in the 60-480 mg/m^2^ dose range after single (day 1) and multiple doses (cycle 1, day 8 [C1D8] and C2D1; Table 4). The mean terminal half-life was 18.5 hours for once-weekly monotherapy, 12.8 hours for twice-weekly monotherapy, and 14.3 hours for combination therapy. For twice-weekly monotherapy, a 6-patient dose cohort was evaluated at 240 mg/m^2^ with exposures similar to once-weekly dosing, with no evidence of accumulation. Exposures to M6620 were similar when this was administered as monotherapy or combined with carboplatin, indicating no interaction between M6620 and carboplatin.

**TABLE 4.**
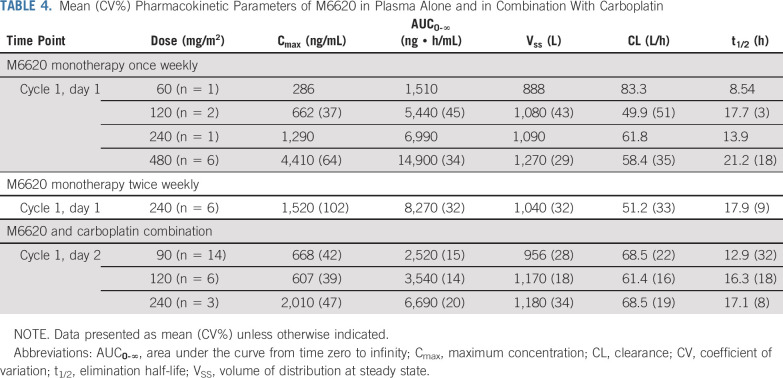
Mean (CV%) Pharmacokinetic Parameters of M6620 in Plasma Alone and in Combination With Carboplatin

### Pharmacodynamic Data

**FIG 2.**
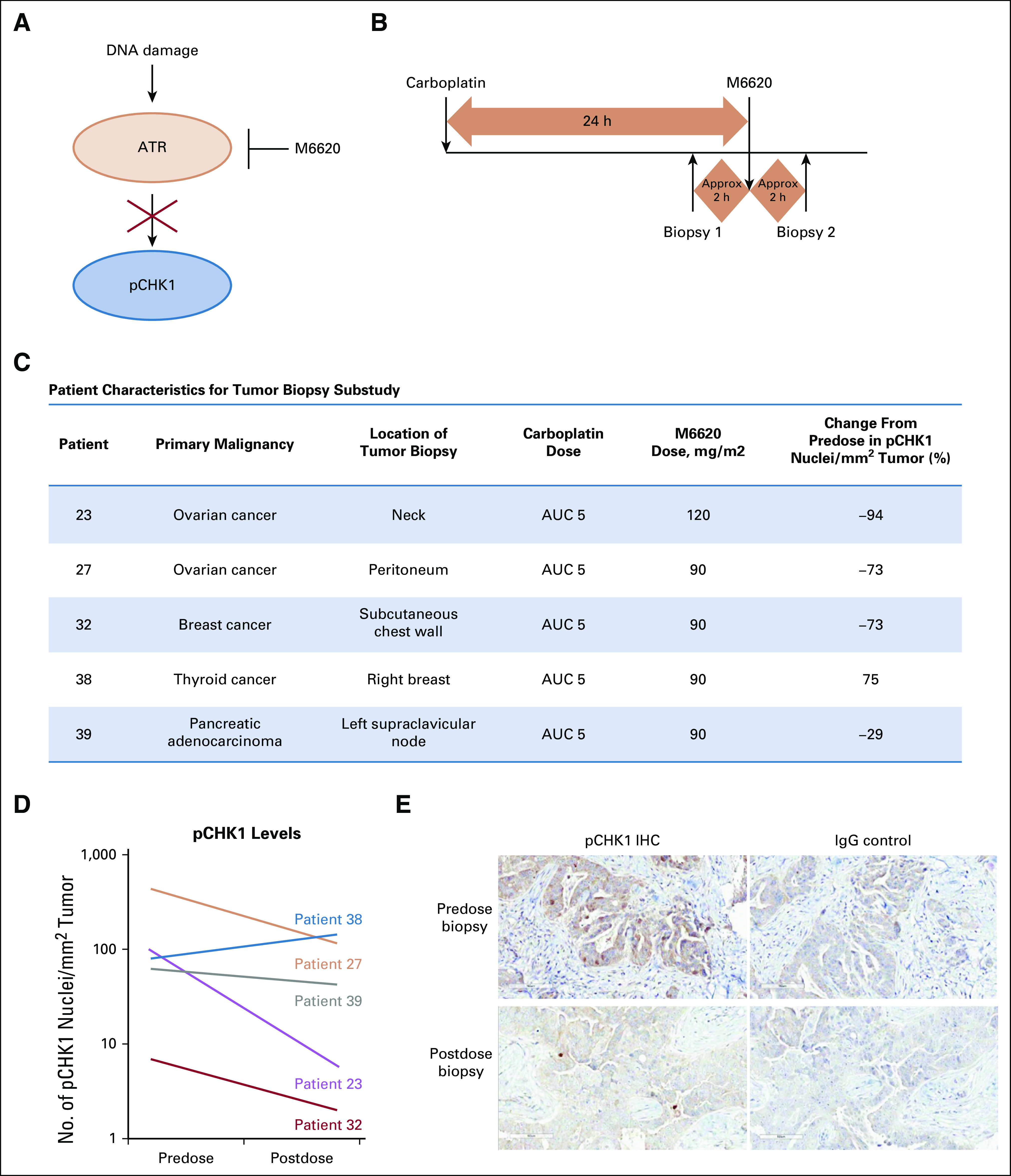
Pharmacodynamic profile of M6620. (A) Ataxia telangiectasia and Rad3-related protein (ATR) pathway schematic. (B) Biopsy schematic. (C) Patient characteristics for biopsy study. (D) Spaghetti plot. (E) Phosphorylated checkpoint kinase 1 (pCHK1) immunohistochemistry (IHC) image from patient 23; predose biopsy (top) and postdose biopsy (bottom), with negative immunoglobulin G (IgG) controls on the right. Approx, approximately; AUC, area under the curve.

To our knowledge, this is the first trial to use precise and rapidly timed paired biopsy pharmacodynamic data to confirm target modulation. Of 12 patients who underwent timed pretreatment and on-treatment tumor biopsies, 5 had both biopsies evaluable (Fig 2). Evaluability was based on tumor content, tumor fixation, and presence of nuclear pCHK1 staining. Of the 5 patients, 4 were treated at the MTD of carboplatin AUC 5 and M6620 90 mg/m^2^, and 1 received carboplatin AUC 5 and M6620 120 mg/m^2^. Overall, a 30%-90% (mean, 67%) reduction in pCHK1 levels was seen in 4 of 5 evaluable biopsy pairs, indicating ATR inhibition by M6620 and supporting the RP2D selection. Additional analyses will be conducted to validate these data.

### Antitumor Responses

**FIG 3.**
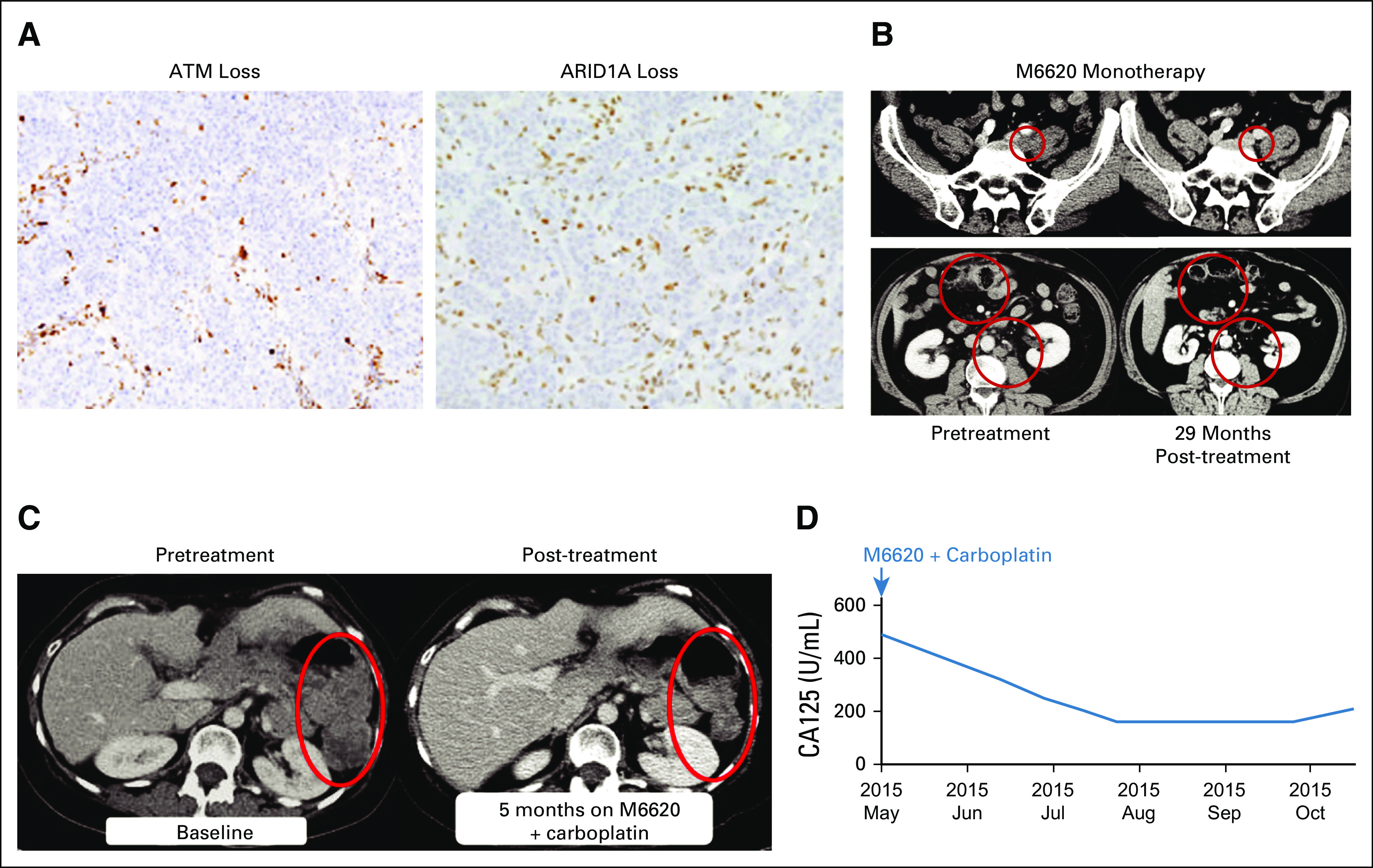
Patient with colorectal cancer who achieved a RECISTv1.1 complete response (CR) to M6620 monotherapy. (A, B) At last assessment, the patient remained in RECISTv1.1 CR with an ongoing progression-free survival of 29 months. At trial baseline, the patient had disease progression in his left common iliac lymph nodes, the largest of which measured 30 mm along the long axis and 18 mm along the short axis (B, top panel). There was also disease progression with worsening peritoneal disease in his anterior abdomen and asymmetric thickening of his transverse colon lateral to surgical clips, in keeping with a local tumor recurrence with transmural infiltration (B, bottom panel). (A) Immunohistochemistry of archived tumor sample showed ataxia-telangiectasia mutated (ATM) loss (left) and AT-rich interaction domain 1A (ARID1A) loss (right). Patient also had other relevant aberrations (see Results section). (B) Computed tomography scans before treatment (left) and 29 months after treatment (right) demonstrated response of left common iliac lymph node and other lesions to single-agent M6620. Top panel shows left common iliac node. Bottom panel shows local recurrence in transverse colon with peritoneal malignant infiltration and left para-aortic nodes. (C, D) Patient with ovarian cancer achieved a RECISTv1.1 partial response to M6620 plus carboplatin combination therapy. (C) Computed tomography scans before (left) and 5 months after therapy (right) showed partial response of left peritoneal disease. (D) There was a corresponding decrease in cancer antigen 125 (CA125) levels, which was a Gynecologic Cancer Intergroup response.

**FIG 4.**
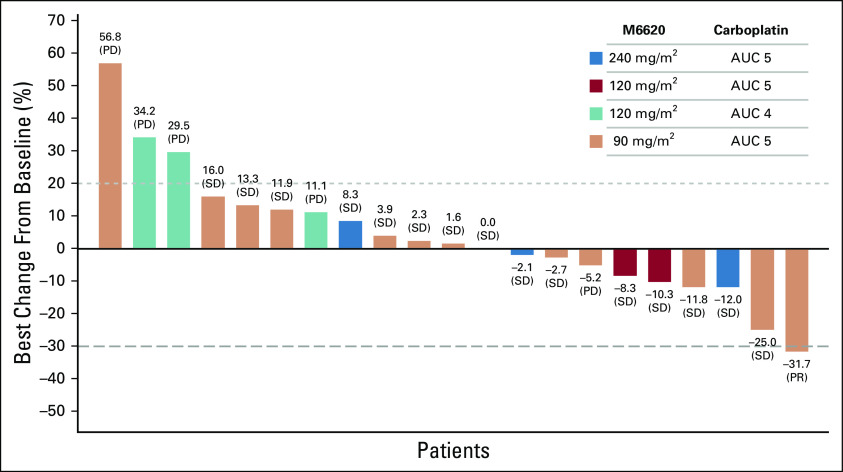
Maximum change from baseline in the sum of target tumor lesion diameters with combination therapy. AUC, area under the curve; PD, progressive disease; PR, partial response; SD, stable disease.

Of 40 patients in the trial, all 17 treated with M6620 monotherapy and 21 of 23 treated with combination therapy were evaluable for efficacy (2 patients discontinued the trial because of infusion reactions and had no efficacy assessments on treatment; therefore, they did not meet prespecified inclusion criteria in efficacy analyses). Response duration is shown in the Data Supplement. One patient achieved a RECISTv1.1 CR with M6620 monotherapy (Fig 3), and 1 achieved a RECISTv1.1 partial response (PR) with combination treatment (Fig 3). Five patients receiving M6620 monotherapy had a best response of RECISTv1.1 stable disease (SD). Fifteen of 21 evaluable patients receiving combination therapy (15 of 23 overall receiving combination therapy) had a best response of RECISTv1.1 SD, with 10 achieving SD for ≥ 4 months, of whom 6 had SD for ≥ 6 months (Fig 4).

Baseline tumor specimens were available for NGS testing from 32 patients (1 with pathogenic *ATM* mutation) and ATM IHC testing from 30 patients (2 with 100% ATM IHC loss; Data Supplement). A patient with advanced gallbladder cancer had a tumor with an *ATM* mutation and 100% ATM IHC loss; this patient achieved a best response of RECISTv1.1 SD but discontinued trial after 11 weeks for early clinical disease progression. A 55-year-old patient with *KRAS* and *BRAF* wild-type advanced colorectal cancer had 100% ATM IHC loss in his tumor, but NGS did not detect an *ATM* mutation. This patient achieved a RECISTv1.1 PR after 4 cycles of once-weekly 60-mg/m^2^ M6620 monotherapy and a RECISTv1.1 CR after 16 cycles and remained in RECISTv1.1 CR with an ongoing progression-free survival (PFS) of 29 months at last assessment (Fig 3). This responder had previously experienced disease progression on multiple lines of chemotherapy, including folinic acid and 5-fluorouracil combined with oxaliplatin, folinic acid and 5-fluorouracil combined with irinotecan, bevacizumab, cetuximab, capecitabine and mitomycin C, and radiotherapy. Mismatch repair deficiency was confirmed by IHC, with loss of MLH1 and PMS2 protein expression. No germline mismatch repair gene alterations were identified. Whole-exome NGS confirmed mismatch repair deficiency with a truncating somatic mutation in *MLH1* and a complex array of other nonsynonymous truncating mutations in DNA repair enzymes, including two heterozygous truncating mutations in *ARID1A* and heterozygous truncating mutations in *CHEK1*, *FANCM*, *RAD50*, *POLD1*, and *FANCP* (*SLX4*) (Data Supplement). Loss of ARID1A protein expression was confirmed by IHC, suggesting that the two *ARID1A* mutations may be in trans, or that mutation of one allele is sufficient for loss at the protein level as observed in other tumor types.^13^ The patient had detectable levels of *ARID1A*, *CHEK1*, *FANCM*, *RAD50*, *PTEN*, *MLH1*, and *MSH6* mutations in circulating free DNA at baseline. The allele frequencies of these mutations declined with M6620 therapy, with *ARID1A* and *FANCM* mutation rates decreasing to undetectable levels in circulating free DNA by C5D1 and *MLH1* mutations becoming undetectable by C9D1.

In the M6620-carboplatin combination cohort, a confirmed RECISTv1.1 PR was observed in a 54-year-old woman with heavily pretreated metastatic high-grade serous ovarian cancer, who was treated at the combination RP2D. Biomarker analyses confirmed germline *BRCA1* Q1111Nfs*5 mutation and *TP53* Y220C missense deleterious somatic mutation (Data Supplement). Her oncology history included initial debulking surgery before 7 lines of different chemotherapy regimens and molecularly targeted agents, including multiple rechallenges with platinum-based chemotherapy before developing platinum-refractory disease on carboplatin-gemcitabine chemotherapy. This patient also received 2 prior lines of PARP inhibitor therapies, achieving a RECISTv1.1 PR lasting 10 months with the first, before developing disease progression. She then received a different PARP inhibitor in combination with an AKT inhibitor, achieving RECISTv1.1 SD with minor tumor regression lasting 5 months, before developing disease progression. She achieved a confirmed RECISTv1.1 PR and Gynecologic Cancer Intergroup CA125 response lasting 6 months with M6620-carboplatin combination therapy at the RP2D (Fig 3). Pharmacodynamic analysis of this patient confirmed on-target ATR inhibition with 73% reduction in pCHK1 expression (Fig 2).

## DISCUSSION

To our knowledge, this report is the first of an ATR inhibitor as monotherapy and combined with carboplatin. The rationale for the trial comes from extensive preclinical studies demonstrating that ATR inhibition lethally sensitizes many cancer cells to DNA-damaging chemotherapies, including carboplatin, and that ATR inhibition can be effective as monotherapy in cancer cells with aberrations that impair alternative repair pathways or that induce high RS.^5-7^

M6620 was initially assessed in this trial as monotherapy on once- and twice-weekly schedules. The drug was well tolerated, with no DLTs on either schedule. The RP2D was established at 240 mg/m^2^ both once and twice weekly. Pharmacokinetics on both schedules were dose proportional, and the observed half-life of M6620 was 12.8-18.5 hours. Although this trial enrolled a heavily pretreated, “all-comer” patient population, 1 patient with advanced colorectal cancer, treated with once-weekly M6620 60 mg/m^2^, achieved a durable RECISTv1.1 CR that was ongoing, with a PFS of 29 months at last assessment. NGS and IHC analyses of the tumor revealed multiple defects in DDR genes and proteins, specifically, heterozygous truncating mutations in *CHK1*, *FANCM*, *RAD50*, *POLD1*, and *FANCP*; compound heterozygous truncating mutations in *ARID1A*; and loss of ATM and ARID1A protein by IHC. Interestingly, many of these genes have been associated with DDR, and, specifically, ATR signaling (*FANCM*, *CHK1*, *ATM*), or have a synthetic lethal relationship with ATR inhibition in vitro and/or in vivo (*POLD1*, *ATM*, *ARID1A*).^6,14-19^ After M6620 treatment, the allele frequency of *ARID1A* and *FANCM* mutations became undetectable, consistent with cells carrying these mutations being sensitive to treatment. These observations and mutations in DDR genes suggest that this exceptional responder had substantial disruption of DNA repair processes, placing an acute reliance on ATR for tumor cell survival.

In combination therapy, M6620 was administered 24 hours after carboplatin administration. This was based on preclinical studies, which demonstrated that maximum efficacy was achieved with administration of ATR inhibitor 12-24 hours after DNA-damaging chemotherapy, coincident with peak accumulation of cells in S-phase and concomitant ATR activation.^8^ Although DLTs within cycle 1 were not observed, mechanism-based myelosuppression resulted in dose delays in carboplatin retreatment in several patients, leading to the RP2D of M6620 90 mg/m^2^ with carboplatin AUC 5. A reduced RP2D for M6620 in combination with carboplatin versus monotherapy is consistent with extensive preclinical studies that demonstrated that nontumor cells undergo transient growth arrest in response to ATR inhibition and DNA damage and that sensitivity to ATR inhibition increases with DNA damage.^6-8^ Importantly, pharmacodynamic activity and RECISTv1.1 PR were demonstrated in the clinic at the RP2D, consistent with the pharmacokinetics and preclinical studies that demonstrated that short periods of transient ATR inhibition (< 2 hours) are sufficient to achieve marked synergy with cytotoxic chemotherapy.^11^ Pharmacokinetic studies confirmed that M6620 exposures were dose dependent, with no evidence of accumulation or interaction with carboplatin.

To gain evidence for ATR activation by carboplatin at the point of M6620 administration (24 hours after carboplatin administration) and to test target engagement at the RP2D, pharmacodynamic studies were undertaken to assess the impact of treatment on CHK1 phosphorylation. Numerous studies have demonstrated that tumor is the optimal tissue to assess pharmacodynamic effects relative to surrogate tissue.^20,21^ We therefore performed a carefully timed paired tumor biopsy study in which patients first underwent biopsy 24 hours after carboplatin chemotherapy, immediately before M6620 administration. This served to assess ATR activation in response to carboplatin (informing if a 24-hour dose delay was appropriate) and as a baseline for the post-M6620 biopsy. This second biopsy was taken within 2 hours of M6620 administration. This tight window is critical, because preclinical studies showed that cells attempt to compensate for ATR inhibition by activating other repair kinases that act on Ser345 of CHK1 (by 2-4 hours).^11^ Five patients had appropriate tumor tissue in their paired biopsy samples. Four patients showed substantial depletion of pCHK1 while receiving treatment with M6620, indicating that the carboplatin combination RP2D/MTD of M6620 90 mg/m^2^ effectively inhibited ATR.

Despite being a heavily pretreated population that was refractory to standard-of-care therapeutic options, 15 of 21 patients eligible for efficacy assessment (15 of 23 overall patients) in the combination arm showed a best response of RECISTv1.1 SD, and 1 treated at the RP2D achieved a RECISTv1.1 PR lasting 6 months. This response was notable because the patient, with advanced high-grade serous ovarian cancer, had disease progression on prior PARP inhibitor therapy twice and was platinum refractory. The patient had a germline *BRCA1* mutation that remained evident on NGS of the pretreatment tumor biopsy and a *TP53* (Y220C) missense mutation. Her persistent *BRCA1* mutation with platinum-refractory and PARP inhibitor–resistant disease (despite initially responding to PARP inhibition), which was responsive to retreatment with platinum in combination with ATR inhibition, is an intriguing finding. It is consistent with the hypothesis that in response to PARP inhibitor therapy or platinum chemotherapy, the tumor acquired a mechanism to activate HR despite retaining BRCA1 deficiency, which can then be blocked with ATR inhibition. It highlights a potential opportunity for ATR inhibitors in restoring platinum sensitivity and treating patients with relapsed *BRCA1/2* mutant disease. A recent study also showed that the combination of M6620 and topotecan was generally well tolerated and demonstrated preliminary antitumor activity in platinum-refractory small-cell lung cancer^22^; a recent phase II study also reported longer PFS with M6620 combined with gemcitabine versus gemcitabine alone in patients with platinum-resistant high-grade serous ovarian cancer.^23^

In summary, M6620 is well-tolerated as monotherapy and in combination with carboplatin. A timed paired tumor biopsy study with the M6620-carboplatin combination supports preclinical data indicating that delayed administration of M6620 is appropriate and that the RP2D is sufficient to inhibit ATR activity in tumors. Two patients achieved RECISTv1.1 responses, 1 with M6620 monotherapy and the other in combination with carboplatin. Multiple trials are ongoing with M6620 and other ATR inhibitors as monotherapy and in rational combinations, such as with DNA-damaging agents.^24^
